# The value of arterial spin labelling perfusion MRI in brain age prediction

**DOI:** 10.1002/hbm.26242

**Published:** 2023-02-27

**Authors:** Mathijs B. J. Dijsselhof, Michelle Barboure, Michael Stritt, Wibeke Nordhøy, Alle Meije Wink, Dani Beck, Lars T. Westlye, James H. Cole, Frederik Barkhof, Henk J. M. M. Mutsaerts, Jan Petr

**Affiliations:** ^1^ Department of Radiology and Nuclear Medicine Amsterdam University Medical Centers, Vrije Universiteit Amsterdam The Netherlands; ^2^ Amsterdam Neuroscience Brain Imaging Amsterdam The Netherlands; ^3^ Mediri GmbH Heidelberg Germany; ^4^ Department of Physics and Computational Radiology, Division of Radiology and Nuclear Medicine Oslo University Hospital Oslo Norway; ^5^ Norwegian Centre for Mental Disorders Research (NORMENT) Oslo University Hospital Oslo Norway; ^6^ Department of Psychology University of Oslo Oslo Norway; ^7^ Department of Psychiatric Research Diakonhjemmet Hospital Oslo Norway; ^8^ KG Jebsen Centre for Neurodevelopmental Disorders University of Oslo Oslo Norway; ^9^ Dementia Research Centre Queen Square Institute of Neurology, UCL London UK; ^10^ Centre for Medical Imaging Computing Computer Science, UCL London UK; ^11^ Queen Square Institute of Neurology and Centre for Medical Image Computing UCL London UK; ^12^ Helmholtz‐Zentrum Dresden‐Rossendorf Institute of Radiopharmaceutical Cancer Research Dresden Germany

**Keywords:** ageing, ASL, brain age, cerebral perfusion, cerebrovascular health, machine learning

## Abstract

Current structural MRI‐based brain age estimates and their difference from chronological age—the brain age gap (BAG)—are limited to late‐stage pathological brain‐tissue changes. The addition of physiological MRI features may detect early‐stage pathological brain alterations and improve brain age prediction. This study investigated the optimal combination of structural and physiological arterial spin labelling (ASL) image features and algorithms. Healthy participants (*n* = 341, age 59.7 ± 14.8 years) were scanned at baseline and after 1.7 ± 0.5 years follow‐up (*n* = 248, mean age 62.4 ± 13.3 years). From 3 T MRI, structural (T1w and FLAIR) volumetric ROI and physiological (ASL) cerebral blood flow (CBF) and spatial coefficient of variation ROI features were constructed. Multiple combinations of features and machine learning algorithms were evaluated using the Mean Absolute Error (MAE). From the best model, longitudinal BAG repeatability and feature importance were assessed. The ElasticNetCV algorithm using T1w + FLAIR+ASL performed best (MAE = 5.0 ± 0.3 years), and better compared with using T1w + FLAIR (MAE = 6.0 ± 0.4 years, *p* < .01). The three most important features were, in descending order, GM CBF, GM/ICV, and WM CBF. Average baseline and follow‐up BAGs were similar (−1.5 ± 6.3 and − 1.1 ± 6.4 years respectively, ICC = 0.85, 95% CI: 0.8–0.9, *p* = .16). The addition of ASL features to structural brain age, combined with the ElasticNetCV algorithm, improved brain age prediction the most, and performed best in a cross‐sectional and repeatability comparison. These findings encourage future studies to explore the value of ASL in brain age in various pathologies.

## Graphical abstract

Current structural MRI‐based brain age estimates and their difference from chronological age are limited to late‐stage pathological brain‐tissue changes. Adding ASL features to structural brain age, combined with the ElasticNetCV algorithm, improved brain age prediction the most and performed best in a cross‐sectional and repeatability comparison. These findings encourage future studies to explore the value of ASL in brain age in various pathologies.

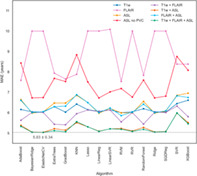

**Abbreviations**
ACA: anterior cerebral artery
AD: Alzheimer's disease
ASL: arterial spin labelling
ATT: arterial transit time
CBF: cerebral blood flow
CoV: spatial coefficient of variation
CSF: cerebrospinal fluid
DKT: Desikan‐Killiany template
GM: grey matter
GPR: Gaussian process regression
ICC: intraclass correlation coefficient
ICV: intracranial volume
KNN: K‐nearest neighbour regression
MAE: mean absolute error
MCA: middle cerebral artery
MCCVs: Monte Carlo cross‐validation simulations
PCA: posterior cerebral artery
PCASL: pseudo‐continuous ASL
PLD: post‐labelling delay
PVC: partial volume correction
RVM: relevance vector machine
RVR: relevance vector regression
SGDreg: stochastic gradient descent regression
SVR: support vector regression
T1w: T1‐weighted
T2w: T2‐weighted
WM: white matter
WMH: white matter hyperintensities

## 1 INTRODUCTION

Accelerated biological ageing is associated with cognitive decline and neurodegenerative disease (Baecker et al., 2021; Franke & Gaser, 2012; Gaser et al., 2013; Hou et al., 2019; Ziegler et al., 2014). Therefore, assessing the impact and role of malleable factors influencing brain ageing and the development of neurodegenerative pathology provides the opportunity for intervention, treatment monitoring, and secondary prevention (Baecker et al., 2021; Franke & Gaser, 2019).

A machine learning method to assess brain ageing is, appropriately named, brain age (Cole & Franke, 2017). Brain age machine learning methods were able to estimate the brain age using information obtained from T1‐weighted (T1w) structural MRI data (Cole et al., 2018). The difference between the predicted and the chronological age—the brain age gap (BAG)*—*can be used to assess deviation from normative ageing trajectories. A larger BAG, therefore, represents a proxy parameter of poorer brain ageing, which is associated with cognitive decline (Biondo et al., 2021; Franke & Gaser, 2019). However, structural brain age makes predictions based on MRI characteristics detailing late‐state changes in brain tissue, therefore focusing only on the morphological effects of ageing and pathology.

The addition of physiological imaging biomarkers may provide a more comprehensive assessment of pathophysiological processes related to ageing. Increasing evidence suggests that declining cerebrovascular health plays a major role in the evolution of cognitive dysfunction (Iadecola & Gottesman, 2019). Although the underlying mechanisms of accelerated cerebrovascular ageing are not yet fully understood, it is implicated in the accelerating cognitive decline present in mild cognitive impairment, vascular dementia, and Alzheimer's disease (AD) (Iturria‐Medina et al., 2016; Plassman et al., 2010) and is associated with future cognitive decline in the cognitively normal population (Pettigrew et al., 2020). Therefore, the addition of vascular MRI biomarkers may complement structural brain age estimation, potentially improving sensitivity to earlier pathological and cognitive changes.

Arterial spin labelling (ASL) is a noninvasive perfusion MRI technique that is capable of measuring changes in cerebral blood flow which has been shown to correlate with the initial stages of cognitive pathology (Iturria‐Medina et al., 2016). Previous results showed that adding ASL‐derived features, ranging from global to subcortical CBF, improved brain age prediction (*R* ^2^ = .77, MAE = 6.4 years) compared with using only global T1‐weighted‐derived features (*R* ^2^ = .72, MAE = 6.9 years) (Rokicki et al., 2021). Furthermore, as brain age estimation utilising ASL‐derived features is rather novel, the optimal machine learning algorithm needs to be determined.

In this study, we investigate the performance of a model combining structural (T1w and FLAIR) and physiological (ASL) image features for predicting brain age, which we refer to as ‘Cerebrovascular brain age’. Using a longitudinal sample of healthy volunteers aged 21 to 95 years, we compared the performance between (1) different imaging features and (2) machine learning algorithms to determine if the addition of ASL‐derived features improves the accuracy of the age prediction. Finally, (3) we estimated the long‐term repeatability of the best‐performing feature set and algorithm.

## 2 MATERIALS AND METHODS

### 2.1 Methods‐study design/data

**FIGURE 1:**
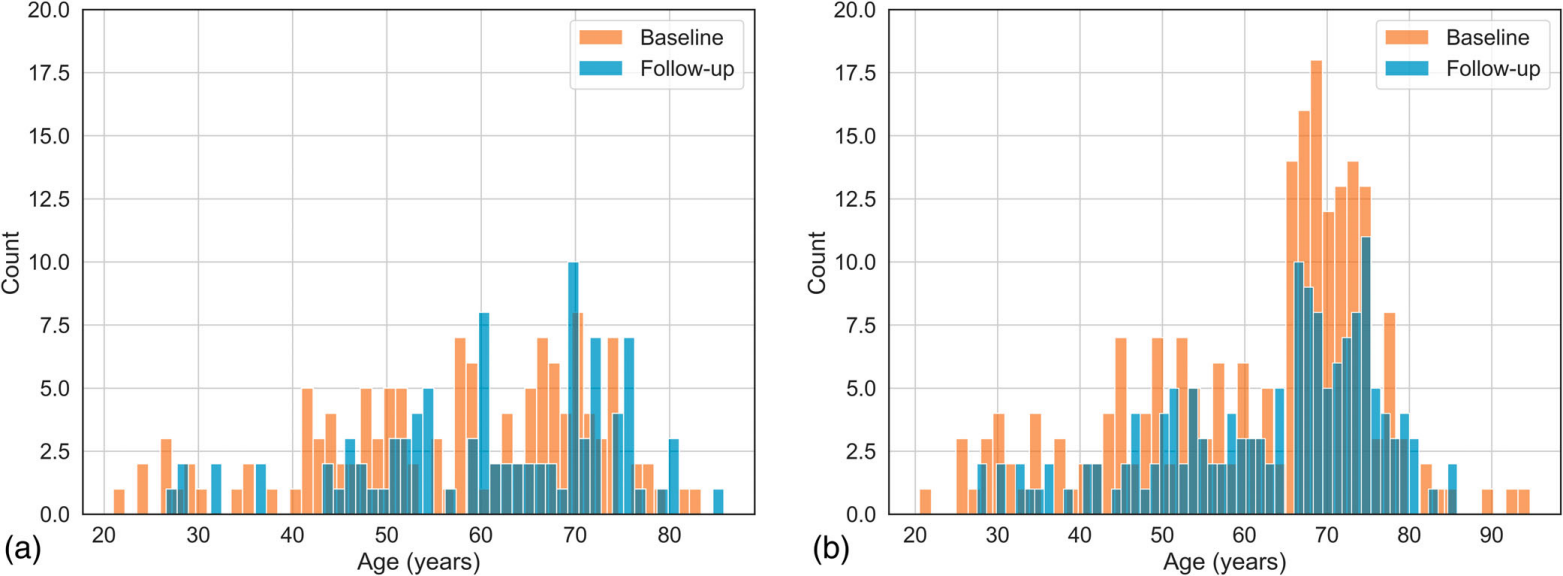
Male (a) and female (b) baseline (orange) and follow‐up (blue) age distributions.

Structural T1w and FLAIR together with ASL and M0 imaging data of healthy participants at baseline (*n* = 341, 38% male, age: 59.7 ± 14.8, range: 21–95 years) and follow‐up after 1.7 ± 0.5 years (*n* = 248, 37.1% male, age: 62.4 ± 13.3, range: 27–86 years) were drawn from the StrokeMRI study (Figure 1) (Beck et al., 2022; Richard et al., 2018). The StrokeMRI study was approved by the Regional Committee for Medical Research Ethics South‐Eastern Norway (2014/64) and the Norwegian Data Inspectorate. Written informed consent was obtained from all participants.

All MRI data were collected using a 3 T MR750 scanner (GE Healthcare), equipped with a 32‐channel head coil. Structural 3D T1w BRAVO images were acquired with the following parameters: repetition time (TR) = 8.2 ms, echo time (TE) = 3.2 ms, inversion time (TI) = 450 ms, flip angle (FA) = 12°, 1.0 × 1.0 × 1.0 mm^3^. Structural 3D FLAIR images were acquired with TR = 8000 ms, TE = 127 ms, TI = 2240 ms, FA = 12°, 1.0 × 1.0 × 1.2 mm^3^. ASL was performed using pseudo‐continuous ASL (PCASL) 3D fast‐spin‐echo interleaved stack‐of‐spirals readout with four background suppression pulses at 3.38, 3.1, 2.6, 1.97 s before the readout, 512 points on 8 spirals with an estimated nominal spatial resolution of 3.8 × 3.8 × 3.0 mm^3^, TE = 11.1 ms, TR = 5025 ms, labelling duration = 1450 ms, post‐labelling delay (PLD) = 2025 ms, number of excitations = 3, accompanied by an M0 image without background suppression or labelling, TR = 2 s.

Image processing was performed with ExploreASL version 1.9.0 (Mutsaerts et al., 2020). Briefly, T1w images were segmented into grey matter (GM), deep white matter (WM), and cerebrospinal fluid (CSF) volumes using the Computational Anatomy Toolbox 12 (Gaser, 2009). White matter hyperintensities (WMHs) were segmented on FLAIR images using the Lesion Segmentation Toolbox, and both volume and count (the number of spatially discrete clusters) were determined (Schmidt et al., 2019). CBF quantification was performed with the recommended single‐compartment model from the perfusion‐weighted and M0 images (Alsop et al., 2015). To create a deep WM mask, the WM partial volume map was thresholded at 60% in ASL native space and subsequently eroding by one voxel to avoid GM contamination, therefore considered to be deep WM (Mutsaerts et al., 2014). Lastly, CBF values were partial‐volume corrected.

All intermediate and final images were rigid‐body registered to the corresponding T1w image, which was then nonlinearly registered to MNI space. Finally, image quality was checked visually. Twenty‐five participants showing ASL‐specific artefacts (movement or arterial transit time‐related) were excluded, resulting in a total of 322 scans at baseline and 242 at follow‐up.

### 2.2 Machine learning feature sets, and algorithms

Image features derived from the T1w, FLAIR, and ASL MRI sequences and their different combinations were used for the brain age training and prediction. To identify the optimal method, features obtained from different anatomical and vascular regions, and different machine learning models were compared in terms of age prediction accuracy, evaluated by mean absolute error (MAE), and coefficient of determination (R^2^). Below, we provide details for all the compared features.

**FIGURE 2:**
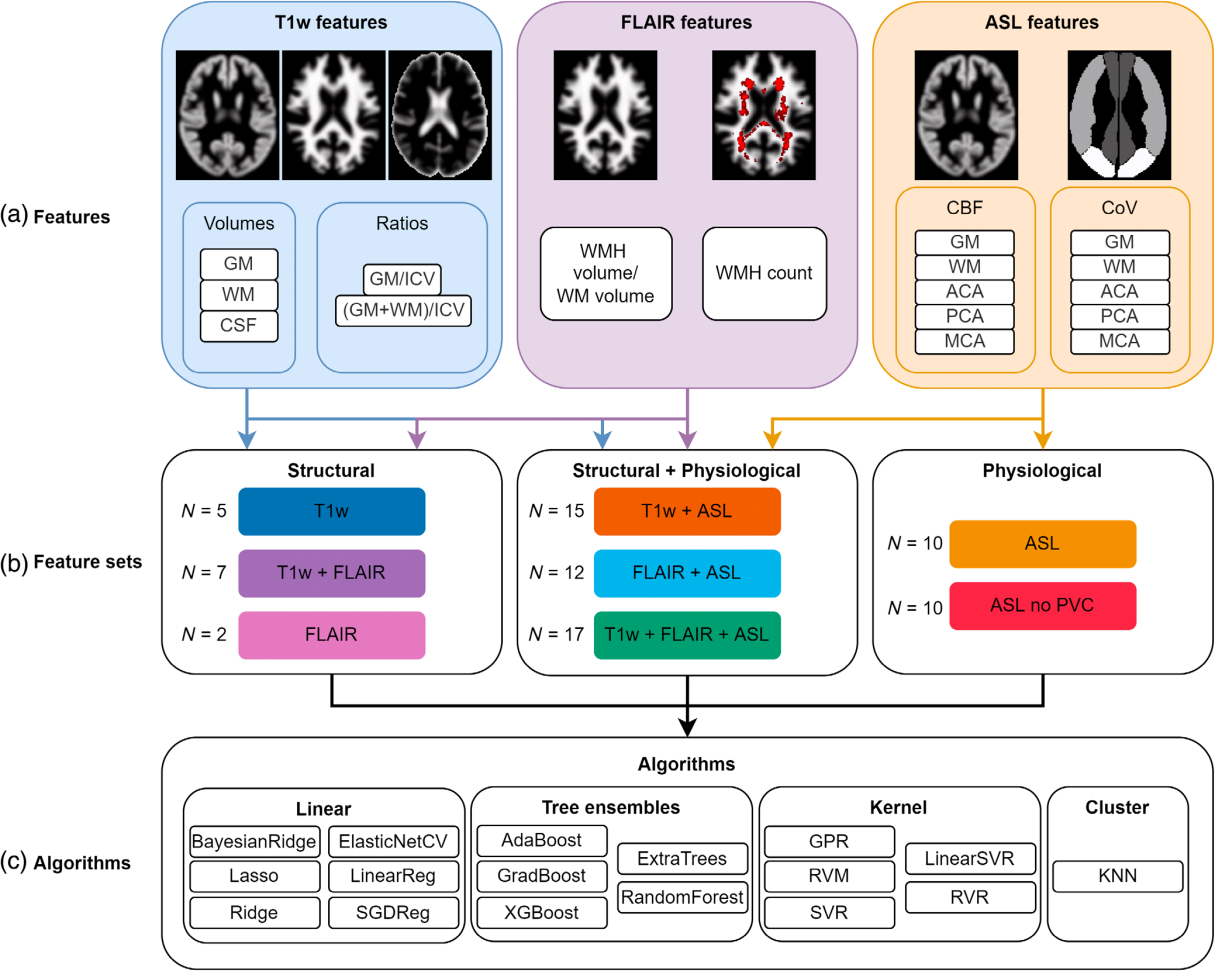
Systematic overview of different features (a), feature sets (b) with *N* showing the number of features, and different algorithms (c) used to predict brain age. ACA, anterior cerebral artery territory; CBF, cerebral blood flow; CoV, coefficient of variation; CSF, cerebrospinal fluid; GM, grey matter; GPR, Gaussian process regression; ICV, intracranial volume; KNN, k‐nearest neighbour regression; MCA, middle cerebral artery territory; PCA, posterior cerebral artery territory; RVM, relevance vector machine; RVR, relevance vector regression; SGDreg, stochastic gradient descent regressor; SVR, support vector regression; WM, deep white matter; WMH, white matter hyperintensity.

Typical structural and physiological image features were extracted from T1w, FLAIR, and ASL images using ExploreASL (Figure 2a). Structural T1w features (Cole, 2020; Cole et al., 2020), were total brain tissue volumes (GM, WM, CSF) and intracranial volume (ICV), and GM/ICV and (GM + WM)/ICV ratios. Here, ICV was defined as the sum of GM, WM, and CSF volumes. Structural FLAIR features (Cole, 2020), were WMH count and volume derived from their respective partial volume maps, where WMH volume was derived as the ratio of WMH to total WM volume. ASL features included regional CBF in total GM, deep WM, vascular territories, and ASL‐derived spatial Coefficient of Variation (CoV) values from the same regions (Mutsaerts et al., 2017). The vascular territories included the regions perfused by the anterior cerebral artery (ACA), middle cerebral artery (MCA), and posterior cerebral artery (PCA) (Tatu et al., 2012). All features were normalised by removing the mean and scaling to unit variance. Various feature sets were constructed by combining all T1w, FLAIR, and ASL features in all possible combinations (Figure 2b).

A variety of linear‐based (simple and regularised), kernel‐based (linear and radial basis), clustering‐based (Minkowski), and tree‐based ensemble (boosting and bagging) algorithms—available within Python packages scikit‐learn version 0.23.1, xgboost library version 1.2.0, and sklearn_rvm library version 0.1.1—were implemented using default settings (Figure 2c).

### 2.3 Training and validation sets

To create data sets for training (training set) and validating (validation set) the brain age models, two training‐validation splits were created; both divided the participants into a 70:30 (*n* = 232, *n* = 100) training and validation sets, respectively:

- **Split I**: to assess feature set and algorithm performance, and keep a sufficiently large sample size, all participants were randomly assigned to either the training or the validation set (resulting in their baseline only or both baseline and follow‐up scans are assigned to the same set).
- **Split II**: to assess longitudinal robustness, the validation set contained participants exclusively having both the baseline and the follow‐up scan. The training set consisted of the remaining participants.

### 2.4 Comparison of machine learning algorithms and image features

For every feature set and algorithm combination (referred to as models), 300 Monte‐Carlo cross‐validation simulations (MCCVs) of Split I were generated, brain age models were trained on each of the training data sets, and then predicted on the corresponding validation data set. The amount of MCCVs was empirically chosen, based on the MAE not improving after a number of simulations. MAE was calculated across the 300 MCCVs as the mean of absolute BAGs—predicted minus chronological brain age—for each participant in the validation data set. Secondly, the proportion of variance (R^2^) was calculated. The average MAE and R^2^ across the validation data set were then compared between every algorithm and feature‐set combination. Next, the best‐performing algorithm per feature set was selected based on the lowest MAE, and compared with the best‐performing algorithm using T1w + FLAIR and ASL‐only features to determine differences between the distribution of MAEs between the best‐performing models, averaged per subject for all MCCVs, then averaged per model. Lastly, the feature importance of the best‐performing model across 300 MCCVs, using Split I, was assessed by determining the weights of the input features (Ishwaran, 2015).

### 2.5 Comparison of ASL regions of interest

ASL‐derived CBF results are commonly averaged across structural regions of interest, for example through the use of the MNI structural atlas (Alisch et al., 2021; Mazziotta et al., 2001). Although effective at detecting structural changes based on anatomically defined areas, vascular changes may not occur in specific anatomical regions but instead in areas fed by larger and smaller branches of the cerebral vasculature.

To test this hypothesis and to add more vascular information to the prediction model, ASL features obtained from the vascular territories were added to the model (Tatu et al., 2012). Desikan‐Killiany template (DKT) derived ASL features, previously used in a study by Rokicki et al. using a similar data set, and vascular‐territory derived features, both using the best‐performing model (ElasticNetCV with T1w + FLAIR + ASL features, Split I) were compared using a paired t‐test. The DKT atlas includes 35 (sub)cortical regions of interest (Desikan et al., 2006; Rokicki et al., 2021).

### 2.6 Comparison with brainageR

We compared our methods with a previously often‐used pre‐trained model called “brainageR, which was trained on GM, WM, and CSF volumes segmented from T1w‐images of 3337 healthy individuals, age: 40.6 ± 21.4 years, age‐range 18–92 years (Biondo et al., 2021). Brain age prediction was compared between the ElasticNetCV algorithm with T1w + FLAIR + ASL features and brainageR using Split II.

### 2.7 Model repeatability

The best‐performing model was selected based on the lowest average MAE and used Split II to predict brain age across 300 MCCVs. To assess model robustness, the repeatability of the BAGs between baseline and follow‐up scans was examined. BAGs were calculated and averaged for all MCCVs for baseline and follow‐up and tested for statistical difference between these two sessions using the paired t‐test and intraclass correlation coefficient (ICC) using a two‐way mixed‐effects model, absolute agreement, and multiple measurements model. The ICC of the best‐performing model was then compared with the two ICCs of the best‐performing algorithm and T1w + FLAIR, and ASL‐only feature sets. Next, average BAG differences between baseline and follow‐up for the best‐performing feature set and algorithm were compared with the best‐performing algorithm using T1w + FLAIR features and ASL features only, using the paired *t*‐test. Lastly, the equality of variances between the same models was compared using Levene's test.

## 3 RESULTS

### 3.1 Comparison of machine learning models

**FIGURE 3:**
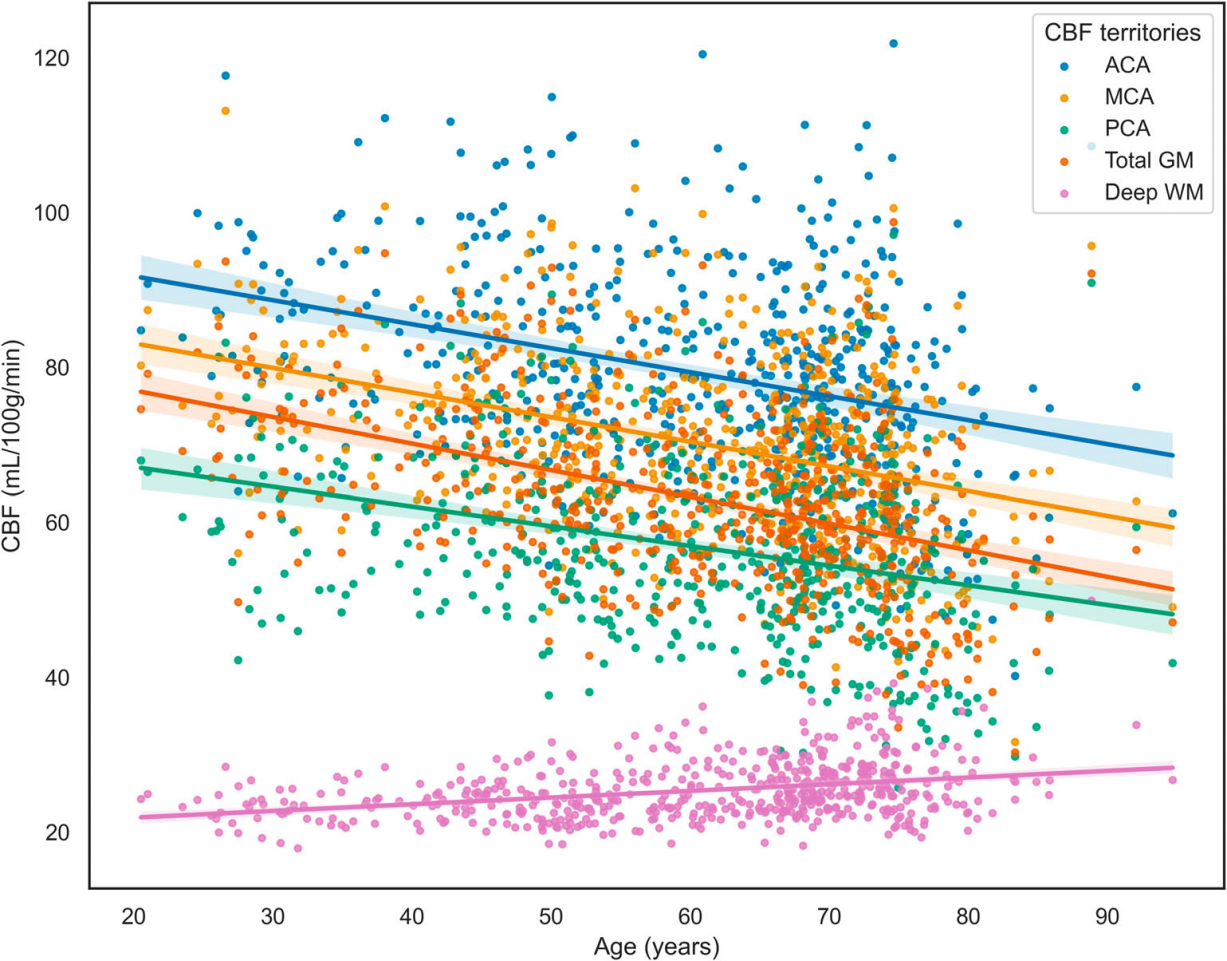
GM, WM, and vascular territory‐based partial volume corrected CBF per age. ACA, anterior cerebral artery; CBF, cerebral blood flow; GM, Grey Matter; MCA, middle cerebral artery; PCA, posterior cerebral artery; WM, deep White Matter.

**FIGURE 4:**
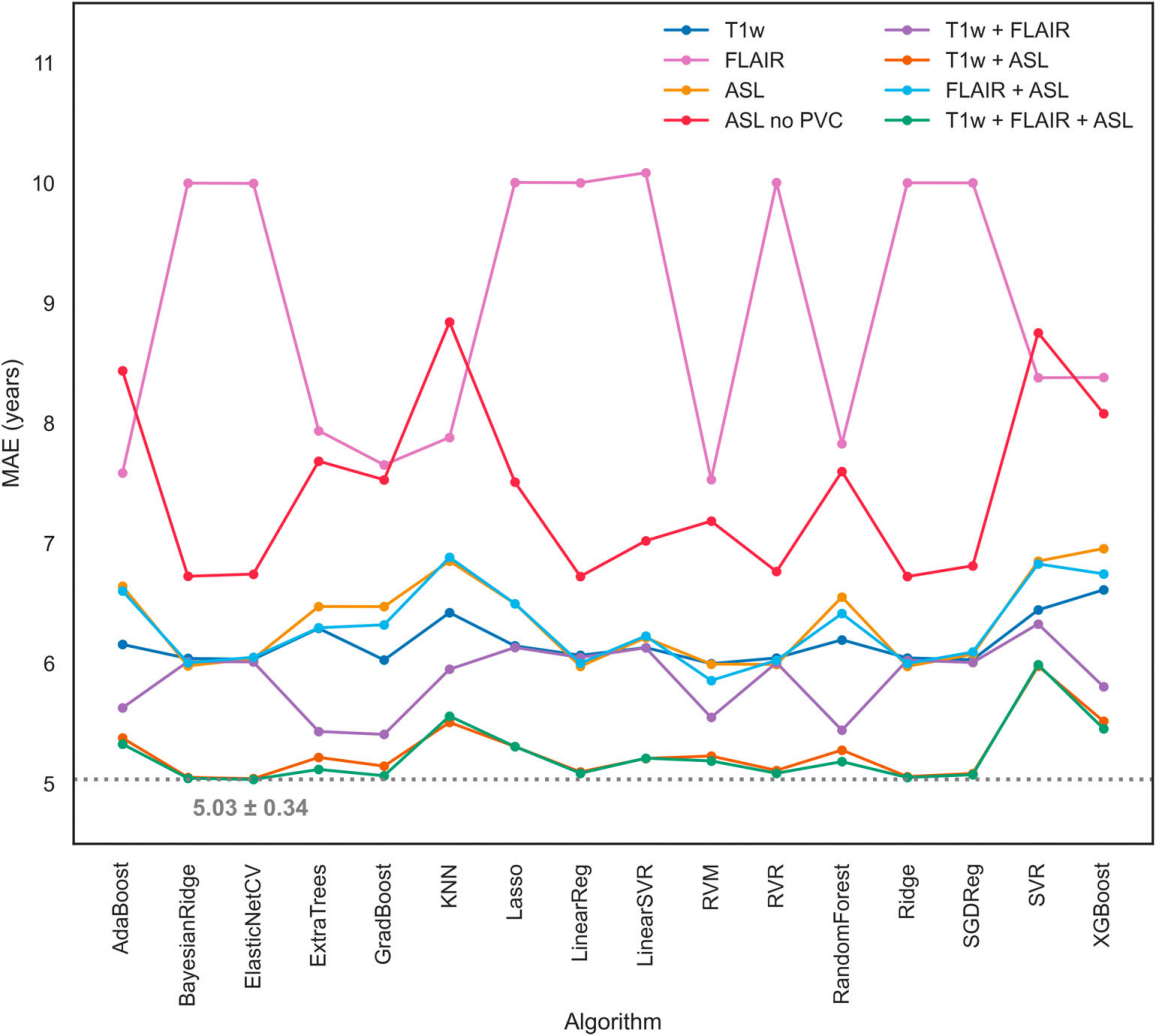
MAE in years for various algorithms and different feature sets after 300 rounds of MCCV. GPR is not shown as it performed poorly. GPR, Gaussian process regression; KNN, k‐nearest neighbour regression; RVM, relevance vector machine; RVR, relevance vector regression; SGDreg, stochastic gradient descent regressor; SVR, support vector regression.

**FIGURE 5:**
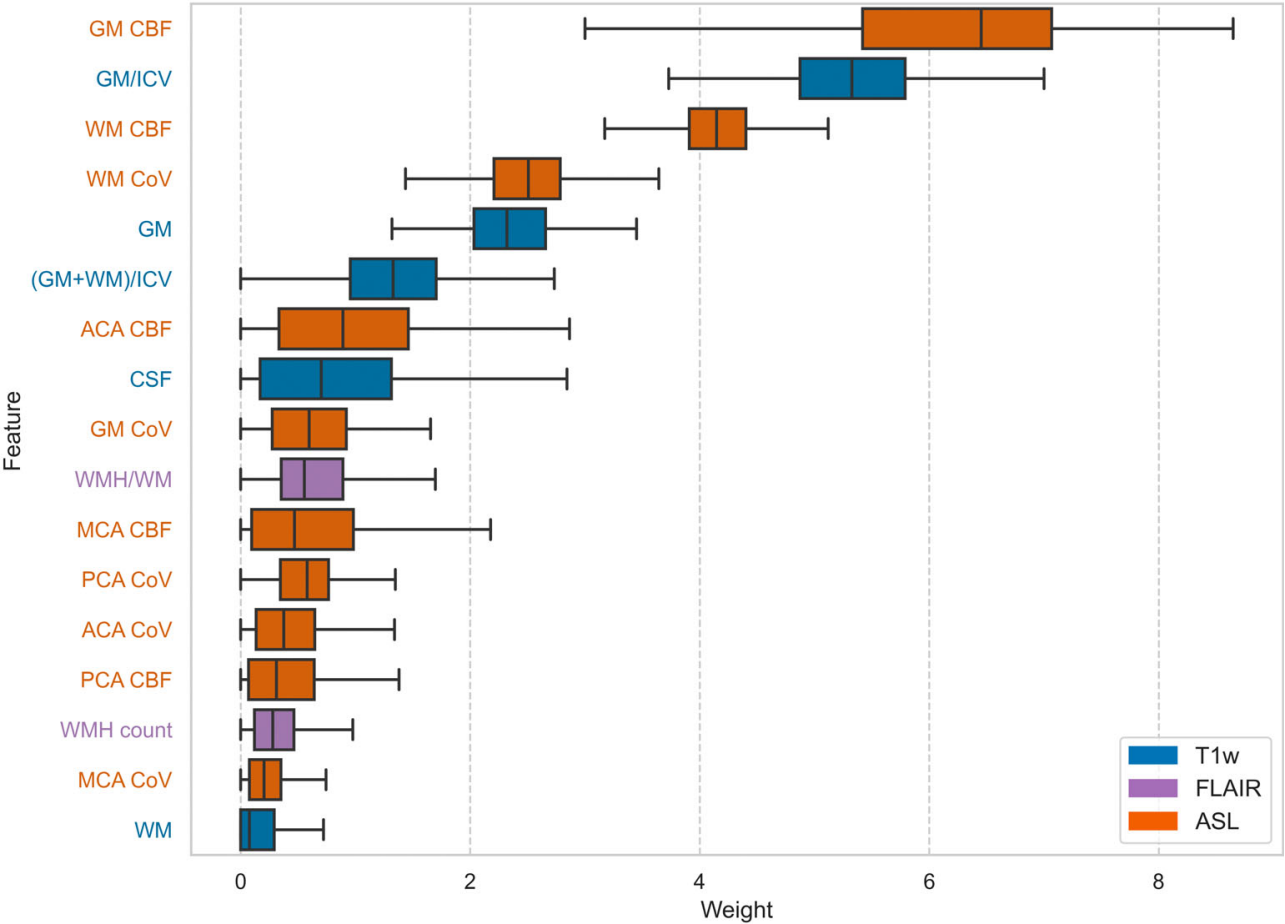
Box plots showing absolute feature weights (arbitrary units) across 300 MCCVs per test subject for the ElasticNetCV algorithm using the T1w + FLAIR + ASL feature set. T1w, FLAIR and ASL features are shown in blue, purple, and orange, respectively. ACA, anterior cerebral artery territory; CBF, cerebral blood flow; CoV, coefficient of variation; CSF, cerebrospinal fluid; GM, grey matter; ICV, intracranial volume; MCA, middle cerebral artery territory; PCA, posterior cerebral artery territory; WM, deep white matter; WMH, white matter hyperintensity.

In the cross‐sectional analysis, GM CBF decreased with 0.37 mL/100 g/min per year (Figure 3). Comparing all models, the ElasticNetCV algorithm with the T1w + FLAIR + ASL feature set performed best (MAE = 5.03 ± 0.34 years, *R* ^2^ = .79 ± .03), which outperformed the T1w + FLAIR feature set (MAE = 6.01 ± 0.39 years, *R* ^2^ = .70 ± .04, *p* < .01) and ASL‐only feature set using the same algorithm (MAE = 6.04 ± 0.39 years, *R* ^2^ = .70 ± .04, *p* < .01) (Figure 4). Compared with the best‐performing model, the GPR algorithm also used by brainageR performed poorly (feature set T1w + FLAIR + ASL, MAE = 8.64 ± 0.62 years, *R* ^2^ = .39 ± .05; data not shown in the figures). Comparing the best model performance per feature set, the ElasticNetCV and T1w + FLAIR + ASL model (MAE = 5.05 ± 3.95 years) significantly outperformed the GradBoost algorithm using T1w + FLAIR (MAE = 5.4 ± 3.93 years, *p* = .01) features and Ridge algorithm using ASL‐only features (MAE = 6.03 ± 4.64 years, *p* < .01). MAE and *R* ^2^ scores for all the remaining feature sets and algorithms are shown in Tables S1 and S2. The three most important features, from highest to lowest absolute average weights, were GM CBF (6.2 ± 1.18), GM/ICV (5.34 ± 0.6), and WM CBF (4.16 ± 0.36; Figure 5).

### 3.2 Vascular territorial and structural ASL features

The model using vascular‐territory‐derived ASL features (MAE = 5.03 ± 0.34) performed better than using DKT‐derived ASL features (MAE =5.28 ± 0.35 years, *p* < .01).

### 3.3 Comparison with brainageR

**FIGURE 6:**
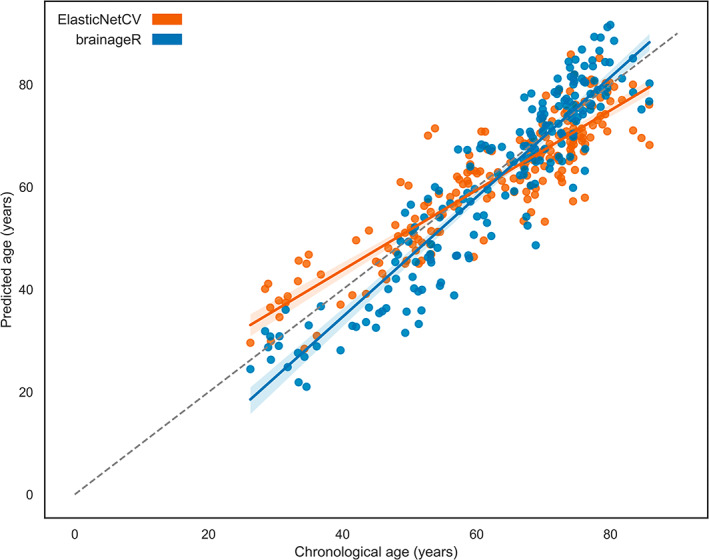
Brain age predictions for our best‐performing model (ElasticNetCV, T1w + FLAIR + ASL) model (orange) and the brainageR (blue) model.

The ElasticNetCV algorithm with T1w + FLAIR + ASL features (MAE = 5.07 ± 4.01 years) performed slightly better than brainageR (MAE = 5.56 ± 4.33 years), although not statistically significantly different (*p* = .11). Lastly, brainageR showed an opposite estimation bias to the ElasticNetCV model (Figure 6).

### 3.4 Repeatability in follow‐up data

**FIGURE 7:**
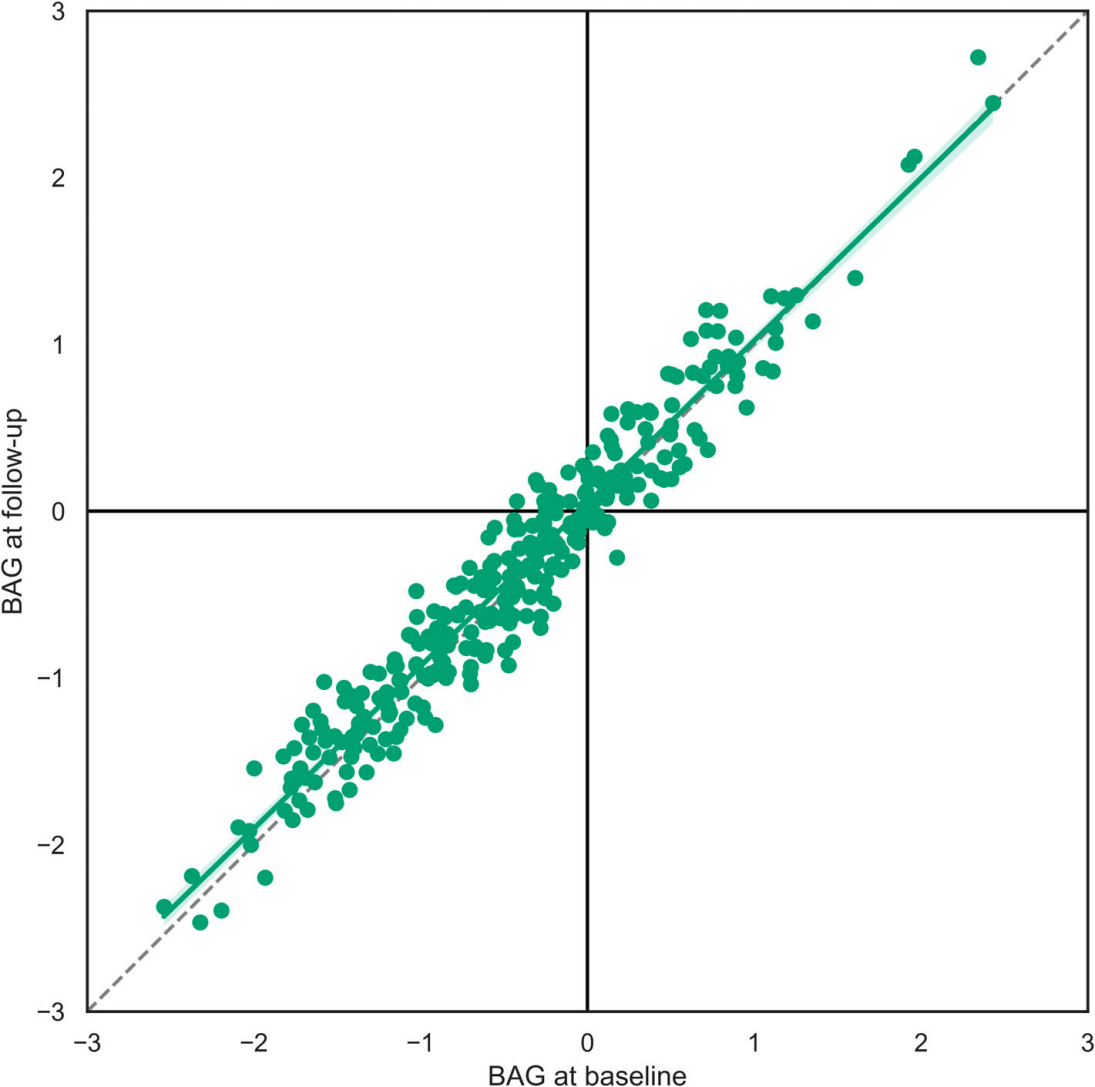
BAGs (predicted age minus chronological age), averaged per MCCV, at baseline and follow‐up sessions obtained with the ElasticNetCV algorithm using the T1w + FLAIR + ASL features. The dotted line represents the line of identity.

The best‐performing model, the ElasticNetCV algorithm with T1w + FLAIR + ASL features, average baseline, and follow‐up BAGs were similar, respectively −0.44 ± 0.85 and −0.39 ± 0.86 years, ICC = 0.97 (95% CI: 0.96–0.98), *p* < .01 (Figure 7). ICC for the ElasticNetCV algorithm using either T1w + FLAIR or ASL‐only features were respectively 0.98 (95% CI: 0.97–0.98) and 0.98 (95% CI: 0.97–0.98).

Longitudinal stability of the average BAGs (the average difference of BAGs between baseline and follow‐up) was similar 0.06 ± 0.21 years) between the T1w + FLAIR + ASL feature set and the T1w + FLAIR feature set (0.05 ± 0.20 years, *p* = .23), and between the T1w + FLAIR + ASL feature set and the ASL‐only feature set (0.04 ± 0.21, *p* = .16). The average standard deviations of the longitudinal BAG stability were lower in the T1w + FLAIR + ASL feature set (2.49 ± 0.7 years) than in both the T1w + FLAIR feature set (2.70 ± 0.73 years, *p* < .01) and the ASL‐only feature set (2.73 ± 0.71 years, *p* < .01). The variances of the BAGs did not differ between T1w + FLAIR + ASL (0.04 years) and T1w + FLAIR feature sets (0.04 years, *p* = .13) or ASL‐only feature sets (0.04 years, *p* = .78).

## 4 DISCUSSION

The main findings of this study were as follows. The added value of ASL was observed by showing that the ElasticNetCV algorithm with the combined multi‐modality feature set (T1w + FLAIR + ASL) had the lowest MAE and highest R^2^, outperforming T1w + FLAIR. Furthermore, ASL features showed high importance in brain age prediction. Lastly, longitudinal consistency and repeatability of brain age predictions were demonstrated for the full set of features and for T1w + FLAIR and ASL feature sets alone.

### 4.1 Feature sets

Comparing structural and physiological feature sets separately, T1w features performed best, ASL intermediate, and FLAIR worst, which is in line with other literature (Cole, 2020; Rokicki et al., 2021). Following this trend, the best‐performing algorithm (ElasticNetCV) using combined features was the T1w + FLAIR + ASL model, followed by the T1w + ASL model, with the T1w + FLAIR model performing worse.

This suggests that both ASL, and FLAIR in a lesser sense, add value to brain age prediction based on T1w features, supporting the idea that these modalities reflect different mechanisms of brain ageing. The worse performance when using FLAIR‐only or FLAIR combined feature sets could be explained by vascular lesions only starting at a late age. In other words, FLAIR images may appear similar across ages except for relatively high age. It is assumed that in healthy ageing, brain volume decline measured by T1w and physiological changes measured by FLAIR and ASL follow a similar pattern on average. However, because of a relatively low prevalence of WMH lesions in a healthy population, FLAIR might be less sensitive to detect these changes and transform them into brain age predictions. Therefore, the performance of FLAIR in brain age models might differ in pathologies associated with WMHs and aberrant cerebral perfusion, such as vascular dementia. FLAIR features have been shown to correlate with structural change, cognitive decline, and Alzheimer's disease (AD) (Haller et al., 2013; Prins & Scheltens, 2015).

Although ASL features perform better than FLAIR, T1w features outperform all other single‐modality feature sets. ASL‐measured CBF changes have been correlated with cognitive decline and AD (Iturria‐Medina et al., 2016). In such diseases, CBF and WMH changes might precede volumetric T1w changes. This might compensate for the higher instrumental and physiological variability of ASL, as appreciated in the large spread of feature importance per MCCVs, and lower prevalence of WMH lesions and lead to a higher prognostic value of cerebrovascular brain age in early disease stages. However, to investigate what information cerebrovascular brain age models provide in cognitively declining or fully impaired cohorts (such as AD), and to disentangle the influences and staging of FLAIR and ASL‐measured brain changes upon cognitive decline, training in a healthy control data set using T1w + ASL or T1w + FLAIR features can be a start.

### 4.2 ASL features

Diving deeper into the role of ASL features in brain age prediction can provide insight into the role of cerebrovascular change in healthy ageing. Interestingly, the third most important ASL feature was deep WM spatial CoV, which is a surrogate of arterial transit time (ATT), which has been coined as a potential surrogate marker of cerebrovascular health (Mutsaerts et al., 2017), This is in line with previous reports of ATT prolongation in healthy ageing (Dai et al., 2017). Spatial CoV in WM with longer ATT than GM might be even more sensitive to ATT changes (Keil et al., 2018). Although the spatial CoV of WM only looks at the distribution of ASL signal, it still remains unclear if the SNR in WM is high enough. To overcome this possible limitation in the future, the vascular territories used in this study could be divided into proximal, medial, and distal regions of interest. The CBF differences with respect to their distance from the labelling plane may reflect the arrival of the label and act as a surrogate of ATT, making deep WM spatial CoV redundant. Furthermore, pathological accelerated ageing may include more complex regional ATT changes than global ATT prolongation seen in healthy ageing. Therefore, feature importance should also be determined for multi‐PLD ASL that provides voxelwise ATT estimates and ATT‐corrected CBF quantification. The relevance of cerebrovascular brain age prediction also has to be validated in cognitively or (cerebro)vascularly impaired cohorts with respect to disease severity. Following WM CoV in feature importance, the ACA CBF feature provides relatively more information on ageing compared with the remaining regions. This is in accordance to the literature, which show the anterior region correlating most (negatively) with age (Lee et al., 2009; Zhang et al., 2018). Although the remaining regions do not account for a high feature importance, it is important to retain them as pathological developments, for example, parietal atrophy in AD, are mostly local instead of generalised across the brain.

By removing the effects of partial tissue volumes upon ASL features, CBF and CoV features showed a large improvement in MAE as compared with nonpartial‐volume‐corrected features. This is an interesting observation as PV effects were believed to overestimate the ASL‐measured CBF decline with age by volume loss changes intensifying the pure GM‐CBF changes (Chappell et al., 2021). Our current findings suggest that PVC, even when used together with structural features, might increase rather than decrease sensitivity to age‐related changes in ASL and potentially also to earlier physiological pathological developments. However, ASL is very sensitive to physiological fluctuations, for example, caffeine intake changes CBF significantly in grey matter, and may therefore complicate brain age prediction (Vidyasagar et al., 2013). This physiological variability may explain why (MacDonald et al., 2020; Rokicki et al., 2021), CBF in itself is not very accurate to predict brain age (MacDonald et al., 2020; Rokicki et al., 2021), while the use of PV‐corrected ASL may increase the accuracy of brain age prediction when combined with other modalities.

### 4.3 Vascular territorial and structural ASL features

Interestingly, using fewer features aimed at vascular regions instead of a large number of anatomical regions slightly improved brain age prediction. This might imply healthy ageing happens more uniformly across the brain and a larger amount of smaller ASL features does not improve prediction, or that a bigger training data set is required. However, pathologically accelerated ageing might occur at a smaller local level and therefore, model performance in pathological cohorts should be investigated further.

### 4.4 Algorithms

The best model consisted of the T1w + FLAIR + ASL feature set and ElasticNetCV algorithm, suggesting this algorithm is most suitable for brain age estimation using multiple and different modalities. However, when sufficiently rich feature sets are used performances become reasonably stable across all algorithms. This suggests feature selection is of more importance than algorithm choice. The best‐performing algorithm on average across different feature sets was the RVM. Relevance vector regression is an algorithm commonly used in brain age estimation and also utilised inside the RVM package (Franke & Gaser, 2019; Tipping, 1999). GPR performs worst in all feature sets. Contrary to these results, GPR combined with structural features performed well in previous applications to determine brain age, however, this has been performed on far larger data sets (*n* = 2001) (Cole et al., 2017). The poor performance of GPR in this data set might be explained by a limited number of features and GPR performance improved drastically when combinations of multiple features were used, instead of only structural (T1w and FLAIR) features.

### 4.5 Comparison with brainageR

The nonsignificant difference in BAG predictions between the best‐performing model and brainageR might be explained by the large difference in training data present. Furthermore, as Split II was used, results may vary for multiple runs. Secondly, our model showed an opposite prediction bias compared with the brainageR model. Although the brainageR model did not correct for this (Biondo et al., 2021), the overestimation at younger ages and underestimation at younger ages of our model is in line with the literature and can be corrected for with different approaches (de Lange & Cole, 2020).

### 4.6 Repeatability

Previously, most brain age methodological studies and applications focused on improving the cross‐sectional age estimation accuracy. However, individual anatomical and physiological differences together with the uncertainty of the MRI measurements set a limit on the theoretically achievable prediction accuracy; and at some point, it becomes questionable if a lower MAE is desirable—because instrumental variability is removed—or if even higher MAE can improve brain age's performance in disease—because meaningful physiological variability is added. Within‐subject longitudinal brain age changes, as a proxy for brain age repeatability, assuming the intra‐individual ageing is negligible compared with the inter‐individual age differences, take the inter‐individual variations out of the equation and might therefore achieve more accurate predictions of age changes across time both for healthy participants, and have accrued interest lately (Aamodt et al., 2022; Franke & Gaser, 2019; Gautherot et al., 2021).

The combined feature set showed similar averaged BAGs, but less averaged variability in individual age gaps across time than the T1w + FLAIR feature set. This suggests that the model's robustness in longitudinal assessment and the detection of subtle pathological changes is increased compared with the T1w + FLAIR and ASL‐only models.

Compared with the ASL‐only feature set, the combined feature set had on average similar BAGs on both sessions, but a significantly lower variability. This implies that physiological changes in healthy ageing are stable in the long‐term, however, subject to short‐term fluctuations. However, this might also partly be due to ASL MRI's sensitivity to physiological change by itself. This, however, needs to be confirmed in an independent data set containing healthy controls and patients.

Although differences in BAGs between baseline and follow‐up were significant and did not show high variance, individual BAGs did show high variance for a single run (Figure 6). This further confirms a prediction bias is present in our models, as discussed in Section 4.5, and should be investigated and compensated for in the future. To further assess longitudinal repeatability in healthy ageing adults, and in order to compare pathological developments on an individual level, further studies are recommended, including multiple follow‐ups and considering a range of lifestyle, health, and environmental variables to determine within and between participant changes in predicted ages (Aamodt et al., 2022; Høgestøl et al., 2022).

### 4.7 Limitations and future directions

Our limitations include the relatively small, single‐site sample, reducing generalisability of the trained model. The age range of the StrokeMRI sample included subjects was large (21–95 years) but were not represented equally. To improve both model prediction accuracy, generalisability, and repeatability, future efforts could be aimed at increasing the sample size by including multiple (longitudinal) cohorts. Although measurement reproducibility of ASL MRI is high (Baas et al., 2021; Mutsaerts et al., 2015), care should be taken when combining multiple cohorts differing in MRI vendor or ASL sequence parameters in order to maintain high reproducibility in brain age prediction.

Furthermore, algorithm parameters were not optimised for this specific data set. This will have adverse effects on brain age prediction, however, it will also increase applicability and generalisability when comparing predicting brain age across multiple cohorts.

In the comparison between brainageR and our best‐performing model, training of our model in Split II has been performed only once with randomisation in subject allocation, therefore results will differ per run and might not be representative of the average performance.

Current features used were based on literature and experience. The best‐performing model (ElasticNetCV with T1w + FLAIR + ASL features), was overestimated at a younger age, while it was underestimated at an older age. While a limitation, this is a common trend in predicting brain age prediction and in‐line with similar with other studies showing similar results, bias correction can be performed to correct for this (de Lange & Cole, 2020).

Further investigation into the use of WM CBF division and upcoming WMH features (based on location and morphology), and additional structural features (thickness and surface) might improve brain age estimation further (Bauer et al., 2021; Bethlehem et al., 2022; Gunning‐Dixon et al., 2009; Lombardi et al., 2020). Other normative modelling methods, for example, by Bethlehem et al., offer different approaches to achieve the same goal in determining healthy brain ageing patterns and pathological deviations. While the methods by Bethlehem et al. utilise single‐modality data and single‐feature nonlinear models linearly combined to determine pathological deviations, multi‐modality machine learning methods such as brain age uses more advanced statistical models while also determining the importance of the features (e.g., GM and WM volume) for predicting normal ageing trajectories, which might provide more accurate measurements of deviation in pathology. Further study is required to determine if both methods should be combined to utilise the nonlinear models of brain charts and feature importance determination of machine learning methods to estimate normal ageing trajectories and pathology (Bethlehem et al., 2022).

## 5 CONCLUSION

We coined cerebrovascular brain age as the combination of structural and physiological features for brain age prediction. The ElasticNetCV algorithm combined with T1w + FLAIR + ASL gave the most accurate brain age prediction, suggesting that the addition of physiological features in the form of ASL improves brain age estimation. This model also proved robust in longitudinal repeatability, showing less variance compared with using structural or ASL features alone. However, results showed that algorithm choice is less important than feature selection. Further improvements in brain age estimation may be achieved by increasing the training set by combining multiple cohorts and through the use of other structural and ASL‐derived physiological features.

## CONFLICT OF INTEREST STATEMENT

The authors declare no conflict of interest. Frederik Barkhof discloses the following: steering committee or iDMC member for Biogen, Merck, Roche, EISAI, and Prothena; consultant for Roche, Biogen, Merck, IXICO, Jansen, Combinostics; research agreements with Merck, Biogen, GE Healthcare, Roche; co‐founder and shareholder of Queen Square Analytics LTD.

## Supporting information


**Data S1:** Supporting Information

## Data Availability

Due to ethical and data security issues related to the sensitive nature of the data, we can not share the data without specific IRB approval and data use agreements with the relevant institution. More information can be obtained from Lars T. Westlye (l.t.westlye@psykologi.uio.no).
