# Supplementary material for: The value of arterial spin labelling perfusion MRI in brain age prediction
Source: Hum Brain Mapp. 2023 Feb 27;44(7):2754–66. doi: 10.1002/hbm.26242 (PMC10089088; doi:10.1002/hbm.26242)
Supplement: Supplementary file 1 — Data S1: Supporting Information [file HBM-44-2754-s001.docx]

Supplementary materials

| **Algorithm** | **T1w** | **FLAIR** | **ASL** | **ASL no PVC** | **T1w + FLAIR** | **T1w + ASL** | **FLAIR + ASL** | **T1w + FLAIR + ASL** | **Average** |
| --- | --- | --- | --- | --- | --- | --- | --- | --- | --- |
| AdaBoost | 6.16 ± 0.42 | 7.59 ± 0.48 | 6.64 ± 0.44 | 8.43 ± 0.56 | 5.63 ± 0.39 | 5.38 ± 0.34 | 6.60 ± 0.42 | 5.33 ± 0.34 | 6.47 ± 0.42 |
| BayesianRidge | 6.04 ± 0.39 | 9.99 ± 0.55 | 5.98 ± 0.39 | 6.73 ± 0.46 | 6.02 ± 0.39 | 5.05 ± 0.35 | 6.00 ± 0.39 | 5.04 ± 0.34 | 6.36 ± 0.41 |
| ElasticNetCV | 6.03 ± 0.39 | 9.99 ± 0.56 | 6.04 ± 0.39 | 6.75 ± 0.47 | 6.01 ± 0.39 | **5.04 ± 0.35** | 6.05 ± 0.39 | **5.03 ± 0.34** | 6.37 ± 0.41 |
| ExtraTrees | 6.29 ± 0.39 | 7.94 ± 0.49 | 6.47 ± 0.41 | 7.69 ± 0.51 | 5.43 ± 0.38 | 5.22 ± 0.34 | 6.3 ± 0.40 | 5.12 ± 0.32 | 6.31 ± 0.41 |
| GradBoost | 6.03 ± 0.38 | 7.65 ± 0.46 | 6.47 ± 0.39 | 7.57 ± 0.53 | **5.41 ± 0.37** | 5.14 ± 0.34 | 6.32 ± 0.38 | 5.06 ± 0.34 | 6.21 ± 0.40 |
| KNN | 6.42 ± 0.41 | 7.88 ± 0.50 | 6.85 ± 0.42 | 8.85 ± 0.53 | 5.95 ± 0.39 | 5.51 ± 0.33 | 6.88 ± 0.42 | 5.56 ± 0.36 | 6.74 ± 0.42 |
| LinearReg | 6.07 ± 0.39 | 10.00 ± 0.56 | 5.98 ± 0.39 | 6.73 ± 0.45 | 6.05 ± 0.39 | 5.10 ± 0.34 | 6.00 ± 0.39 | 5.09 ± 0.34 | 6.38 ± 0.41 |
| LinearSVR | 6.13 ± 0.40 | 10.09 ± 0.56 | 6.21 ± 0.43 | 7.01 ± 0.49 | 6.13 ± 0.40 | 5.21 ± 0.37 | 6.23 ± 0.43 | 5.21 ± 0.35 | 6.53 ± 0.43 |
| RandomForest | 6.2 ± 0.39 | 7.83 ± 0.47 | 6.55 ± 0.42 | 7.59 ± 0.52 | 5.44 ± 0.38 | 5.28 ± 0.35 | 6.42 ± 0.43 | 5.18 ± 0.35 | 6.31 ± 0.41 |
| Ridge | 6.05 ± 0.39 | 10.00 ± 0.55 | **5.97 ± 0.39** | **6.73 ± 0.46** | 6.03 ± 0.39 | 5.06 ± 0.34 | 6.00 ± 0.39 | 5.05 ± 0.34 | 6.36 ± 0.41 |
| RVM | **5.99 ± 0.42** | **7.53 ± 0.45** | 5.99 ± 0.38 | 7.25 ± 0.50 | 5.55 ± 0.40 | 5.23 ± 0.35 | **5.86 ± 0.38** | 5.19 ± 0.37 | **6.07  0.41** |
| RVR | 6.05 ± 0.39 | 10.00 ± 0.55 | 5.99 ± 0.39 | 6.77 ± 0.46 | 6.01 ± 0.39 | 5.11 ± 0.35 | 6.03 ± 0.39 | 5.09 ± 0.34 | 6.38 ± 0.41 |
| SGDReg | 6.03 ± 0.39 | 10.00 ± 0.56 | 6.08 ± 0.39 | 6.82 ± 0.47 | 6.01 ± 0.39 | 5.08 ± 0.35 | 6.09 ± 0.39 | 5.07 ± 0.34 | 6.4 ± 0.41 |
| SVR | 6.45 ± 0.46 | 8.38 ± 0.56 | 6.85 ± 0.49 | 8.78 ± 0.61 | 6.33 ± 0.46 | 5.98 ± 0.45 | 6.83 ± 0.48 | 5.99 ± 0.45 | 6.95 ± 0.50 |
| XGBoost | 6.61 ± 0.42 | 8.38 ± 0.49 | 6.96 ± 0.42 | 8.10 ± 0.53 | 5.81 ± 0.38 | 5.52 ± 0.35 | 6.75 ± 0.40 | 5.46 ± 0.36 | 6.7 ± 0.42 |
| Lasso | 6.15 ± 0.39 | 10.00 ± 0.55 | 6.50 ± 0.44 | 7.52 ± 0.57 | 6.13 ± 0.39 | 5.31 ± 0.38 | 6.5 ± 0.44 | 5.31 ± 0.38 | 6.68 ± 0.44 |
| GPR | 895.1 ± 270.96 | 496.5 ± 494.82 | 8.07 ± 0.43 | 9.24 ± 0.52 | 20.86 ± 3.49 | 8.01 ± 0.56 | 8.13 ± 0.50 | 8.64 ± 0.62 |  |
| Average* | 6.17 ± 0.40 | 8.95 ± 0.52 | 6.35 ± 0.41 | 7.46 ± 0.52 | 5.87 ± 0.39 | 5.87 ± 0.39 | 6.3 ± 0.41 | **5.24 ± 0.35** |  |

**Table 1:** Mean absolute error (MAE) in years per featureset and algorithm. Best algorithms per feature set are shown in bold. Average values per feature set and algorithm exclude GPR (which failed to provide meaningful results in the T1w and FLAIR feature sets with default parameters), to avoid contamination with large values.

| **Algorithm** | **T1w** | **FLAIR** | **ASL** | **ASL no PVC** | **T1w + FLAIR** | **T1w + ASL** | **FLAIR + ASL** | **T1w + FLAIR + ASL** | **Average** |
| --- | --- | --- | --- | --- | --- | --- | --- | --- | --- |
| AdaBoost | 0.68 ± 0.05 | 0.51 ± 0.06 | 0.62 ± 0.05 | 0.42 ± 0.06 | 0.73 ± 0.04 | 0.76 ± 0.04 | 0.63 ± 0.05 | 0.77 ± 0.04 | 0.64 ± 0.05 |
| BayesianRidge | 0.70 ± 0.04 | 0.22 ± 0.05 | 0.71 ± 0.04 | 0.62 ± 0.05 | 0.70 ± 0.04 | 0.79 ± 0.03 | 0.70 ± 0.04 | 0.79 ± 0.03 | 0.65 ± 0.04 |
| ElasticNetCV | **0.70 ± 0.04** | 0.22 ± 0.05 | 0.70 ± 0.04 | 0.62 ± 0.05 | 0.70 ± 0.04 | 0.79 ± 0.03 | 0.70 ± 0.04 | **0.79 ± 0.03** | 0.65 ± 0.04 |
| ExtraTrees | 0.66 ± 0.05 | 0.43 ± 0.07 | 0.64 ± 0.05 | 0.50 ± 0.06 | 0.75 ± 0.04 | 0.77 ± 0.03 | 0.66 ± 0.04 | 0.78 ± 0.03 | 0.65 ± 0.05 |
| GradBoost | 0.69 ± 0.05 | 0.49 ± 0.06 | 0.64 ± 0.05 | 0.51 ± 0.06 | **0.75 ± 0.04** | 0.78 ± 0.03 | 0.66 ± 0.05 | 0.79 ± 0.03 | 0.66 ± 0.05 |
| KNN | 0.65 ± 0.05 | 0.46 ± 0.07 | 0.61 ± 0.05 | 0.37 ± 0.08 | 0.70 ± 0.04 | 0.74 ± 0.03 | 0.61 ± 0.05 | 0.74 ± 0.04 | 0.61 ± 0.05 |
| LinearReg | 0.70 ± 0.04 | 0.22 ± 0.05 | **0.71 ± 0.04** | **0.62 ± 0.05** | 0.70 ± 0.04 | 0.79 ± 0.03 | 0.71 ± 0.04 | 0.79 ± 0.03 | 0.65 ± 0.04 |
| LinearSVR | 0.69 ± 0.05 | 0.21 ± 0.06 | 0.68 ± 0.05 | 0.60 ± 0.06 | 0.69 ± 0.05 | 0.78 ± 0.04 | 0.68 ± 0.05 | 0.78 ± 0.03 | 0.64 ± 0.05 |
| RandomForest | 0.67 ± 0.05 | 0.46 ± 0.07 | 0.63 ± 0.05 | 0.51 ± 0.06 | 0.75 ± 0.04 | 0.77 ± 0.04 | 0.65 ± 0.05 | 0.78 ± 0.03 | 0.65 ± 0.05 |
| Ridge | 0.70 ± 0.04 | 0.22 ± 0.05 | 0.71 ± 0.04 | 0.62 ± 0.05 | 0.70 ± 0.04 | **0.79 ± 0.03** | 0.70 ± 0.04 | 0.79 ± 0.03 | 0.65 ± 0.04 |
| RVM | 0.68 ± 0.05 | **0.52 ± 0.05** | 0.69 ± 0.05 | 0.56 ± 0.06 | 0.73 ± 0.04 | 0.77 ± 0.03 | **0.71 ± 0.04** | 0.77 ± 0.03 | **0.68 ± 0.04** |
| RVR | 0.70 ± 0.04 | 0.22 ± 0.05 | 0.71 ± 0.04 | 0.62 ± 0.05 | 0.70 ± 0.04 | 0.78 ± 0.03 | 0.70 ± 0.04 | 0.78 ± 0.03 | 0.65 ± 0.04 |
| SGDReg | 0.70 ± 0.04 | 0.22 ± 0.05 | 0.70 ± 0.04 | 0.61 ± 0.05 | 0.70 ± 0.04 | 0.78 ± 0.03 | 0.69 ± 0.04 | 0.78 ± 0.03 | 0.65 ± 0.04 |
| SVR | 0.63 ± 0.04 | 0.39 ± 0.05 | 0.59 ± 0.03 | 0.38 ± 0.05 | 0.64 ± 0.04 | 0.68 ± 0.03 | 0.59 ± 0.03 | 0.67 ± 0.03 | 0.57 ± 0.04 |
| XGBoost | 0.63 ± 0.06 | 0.37 ± 0.08 | 0.59 ± 0.06 | 0.43 ± 0.08 | 0.72 ± 0.05 | 0.75 ± 0.04 | 0.61 ± 0.06 | 0.75 ± 0.04 | 0.61 ± 0.06 |
| Lasso | 0.69 ± 0.04 | 0.22 ± 0.04 | 0.64 ± 0.04 | 0.54 ± 0.04 | 0.70 ± 0.04 | 0.77 ± 0.03 | 0.64 ± 0.04 | 0.77 ± 0.03 | 0.62 ± 0.04 |
| GPR | -31744.18 ± 21464.97 | -101578.63 ± 177637.59 | 0.46 ± 0.06 | 0.32 ± 0.08 | -3.93 ± 2.01 | 0.46 ± 0.05 | 0.46 ± 0.06 | 0.39 ± 0.05 |  |
| Average* | 0.68 ± 0.05 | 0.34 ± 0.06 | 0.66 ± 0.04 | 0.53 ± 0.06 | 0.71 ± 0.04 | 0.77 ± 0.03 | 0.67 ± 0.04 | **0.77 ± 0.03** |  |

**Table 2 :** R^2^ per featureset and algorithm. Best algorithms per feature set are shown in bold. Average values per feature set and algorithm exclude GPR (which failed to provide meaningful results in the T1w and FLAIR feature sets with default parameters), to avoid contamination with large values.
